# Does a History of Stroke Affect Outcomes in Non-variceal Upper Gastrointestinal Bleeding?

**DOI:** 10.7759/cureus.89159

**Published:** 2025-07-31

**Authors:** Anupam Gupta, Anudeep Surendranath

**Affiliations:** 1 Surgery, Bangalore Medical College, Bangalore, IND; 2 Surgery, Kempegowda Institute of Medical Science, Bangalore, IND; 3 Neurology, CHI St. Vincent Hot Springs, Hot Springs, USA

**Keywords:** acute upper gastrointestinal bleeding, cute non-variceal bleeding, ischemic stroke, national inpatient sample database, stroke

## Abstract

Background and purpose

Non-variceal upper gastrointestinal (GI) bleeding is a potentially life-threatening condition. This study aimed to evaluate clinical outcomes in patients presenting with non-variceal upper GI bleeding who had a prior history of ischemic stroke.

Methods

The 2021 National Inpatient Sample database was used to identify 259,025 patients diagnosed with non-variceal upper GI bleeding, of whom 1,485 had a documented history of ischemic stroke. Data were analyzed using Stata version 18 (StataCorp LLC, College Station, TX, US) to assess the primary outcome of in-hospital mortality. Secondary outcomes included length of hospital stay, hospitalization cost, and discharge disposition.

Results

Among the 259,025 admissions for non-variceal upper GI bleeding, 1,485 (0.57%) patients had a prior ischemic stroke. The mean age of these patients was 72.35 years, and they had a higher prevalence of comorbidities. A history of ischemic stroke was associated with significantly increased in-hospital mortality (OR 7.51, p < 0.01), a longer hospital stay by an average of 5.68 days (p < 0.01), and a greater need for discharge to a skilled nursing facility (OR 3.30, p < 0.01). No statistically significant associations were found between race, median income, or geographic region and clinical outcomes.

Conclusion

Patients with a history of ischemic stroke presenting with non-variceal upper GI bleeding are typically older and have more comorbidities. Prior stroke is an independent risk factor for increased mortality, longer hospitalization, higher healthcare costs, and a greater likelihood of discharge to a nursing facility in this population.

## Introduction

Stroke remains a leading cause of mortality and significant long-term disability in both the United States and globally [1,2]. Worldwide, the estimated lifetime risk of stroke is approximately 25% among adults aged 25 and older [3]. Advances in early recognition and timely recanalization therapies, such as intravenous thrombolysis and endovascular treatment, have improved stroke-related outcomes [4]. Antithrombotic agents have shown efficacy in both acute and long-term management of ischemic stroke [5]. Common regimens for secondary prevention include aspirin alone or in combination with clopidogrel or oral anticoagulants [5,6].

Upper gastrointestinal (GI) bleeding, defined as bleeding proximal to the ligament of Treitz, occurs at an estimated rate of 48-160 cases per 100,000 adults annually, with a mortality rate of 3.3-4.3 per 10,000 in the United States [7-9]. Upper GI endoscopy is both diagnostic and therapeutic in most cases [8]. Management typically involves intravenous proton pump inhibitors and blood transfusion; patients on anticoagulants may require reversal therapy in life-threatening cases [9-11].

GI bleeding during stroke hospitalization is associated with poorer outcomes and increased mortality [12,13]. Approximately 87% of all strokes are ischemic [14], and 1.5%-8% of stroke patients experience acute GI bleeding during hospitalization. One Japanese cohort reported nearly half of such events occurring within the first week of admission [12-14]. These bleeding episodes are linked to higher mortality, worse neurological recovery, and increased risk of recurrent stroke [13-15].

Several risk factors for ischemic stroke, including older age, male sex, Black race, psychological stress, hypertension, smoking, obesity, poor diet, sedentary lifestyle, alcohol use, hyperlipidemia, certain medications, and psychosocial conditions, may also predispose individuals to upper GI bleeding [13,15].

Managing non-variceal upper GI bleeding often necessitates withholding or reversing antithrombotic therapy and administering blood products. These interventions may conflict with the long-term secondary prevention strategies for ischemic stroke, which depend heavily on continued antithrombotic use [13,16].

This study aimed to evaluate clinical outcomes in patients admitted with non-variceal upper GI bleeding who had a prior history of ischemic stroke.

This work was previously posted as a preprint on the medRxiv server in May 2025.

## Materials and methods

Study design and database description

This study utilized the 2021 National Inpatient Sample (NIS), part of the Healthcare Cost and Utilization Project (HCUP) under the Agency for Healthcare Research and Quality (AHRQ). The NIS is a publicly available, de-identified database representing approximately 20% of all inpatient hospitalizations in the United States. It contains demographic, diagnostic, procedural, and hospital-level information, enabling national estimates of inpatient care. Because the dataset is de-identified, Institutional Review Board approval and informed consent were not required.

Study population

This study included all patients admitted with a principal diagnosis of non-variceal upper GI bleeding, identified using International Classification of Diseases, Tenth Revision (ICD-10) codes (Table 1). Patients were divided into two groups based on the presence or absence of a history of ischemic stroke (ICD-10 codes I63.x, where x represents all subcodes). We compared patient demographics and hospital characteristics between these groups to evaluate differences in clinical presentation and outcomes.

**Table 1:** ICD-10 codes used to identify non-variceal upper gastrointestinal (GI) bleeding

Category	ICD-10 codes
Gastric ulcers with hemorrhage	K25.0, K25.2, K25.4, K25.6
Duodenal ulcers with hemorrhage	K26.0, K26.2, K26.4, K26.6
Peptic ulcers, site unspecified	K27.0, K27.2, K27.4, K27.6
Gastrojejunal ulcers	K28.0, K28.2, K28.4, K28.6
Gastritis and duodenitis with bleeding	K29.01, K29.21, K29.31, K29.41, K29.51, K29.61, K29.71, K29.81, K29.91
Angiodysplasia and Dieulafoy lesion	K31.811, K31.82
Esophagitis and esophageal ulcer with bleeding	K21.01, K20.91, K22.11
Hematemesis	K92.0
Non-specific upper GI hemorrhage	K92.1 (Melena), K92.2 (GI hemorrhage, unspecified), D62 (Acute post-hemorrhagic anemia)
Neoplasms associated with potential bleeding	D13.0–D13.2, C15.3–C15.9, C16.0–C16.9, C17.0
Other esophageal and gastric lesions	K22.10, K20.80, K20.8, K20.9, K25.3, K25.7, K25.9, K31.819, K21.00

Outcomes

The primary outcome of interest in this study was in-hospital mortality, comparing patients with and without a history of ischemic stroke who were admitted with a principal diagnosis of non-variceal upper GI bleeding. Secondary outcomes included total hospital length of stay (LOS), total hospital charges, and discharge disposition, which serve as critical metrics reflecting resource utilization, healthcare burden, and post-hospitalization care needs.

Statistical analysis

Data analysis was performed using Stata version 18 (StataCorp LLC, College Station, TX, US). T-tests were used to calculate p-values for continuous variables, while Fisher’s exact test was applied to categorical and binary variables. To evaluate the association between a history of ischemic stroke and binary outcomes, multivariate logistic regression analysis was conducted, while multivariate linear regression was employed for continuous outcomes. Both models were adjusted for potential confounders to enhance the accuracy and reliability of the results. All tests were two-tailed, and a p-value of <0.05 was considered statistically significant.

## Results

Primary outcome

Patient and Hospital Characteristics

The NIS dataset included 33.3 million observations, of which 259,025 were patients admitted with a principal diagnosis of non-variceal upper GI bleeding. Among these, 1,485 (0.57%) had a comorbid diagnosis of ischemic stroke. Table 2 offers a comprehensive comparison of patient and hospital characteristics for patients with and without an ischemic stroke diagnosis.

**Table 2:** Patient and hospital characteristics

	No ischemic stroke	Ischemic stroke	p-value
Female (%)	120,000 (45.13)	640 (43.1)	0.49
Male(%)	139,025 (53.67)	845 (56.9)	
Mean age (95% confidence interval)	66.55 (66.35-66.75)	72.35 (71.00-73.69)	<0.01
Race/ethnicity (%)			0.07
White	170,000 (67.93)	960 (67.37)	
Black	36,000 (14.53)	240 (16.84)	
Hispanic	27,000 (10.82)	100 (7.02)	
Asian or Pacific Islander	8,410 (3.35)	70 (4.91)	
Native American	2,675 (1.07)	5 (0.35)	
Other	5,795 (2.31)	50 (3.51)	
Median annual income in patient’s zip code, US$, no. (%)			0.21
$1-$51,999	78,000 (30.78)	455 (30.95)	
$52,000-$65,999	65,000 (25.64)	345 (23.47)	
$66,000-$87,999	60,000 (22.92)	425 (28.91)	
$88,000 or more​	50,000 (19.67)	245 (16.67)	
Insurance type, no. (%)			<0.01
Medicare	160,000 (63.56)	1,100 (76.12)	
Medicaid	36,000 (14.37)	130 (9.0)	
Private insurance, including Health Maintenance Organizations (HMOs)	44,000 (17.65)	150 (10.38)	
Self-pay	11,000 (4.42)	65 (4.5)	
Hospital region, no. (%)			0.34
Northeast	42,000 (16.48)	235 (15.82)	
Midwest	57,000 (22.17)	395 (26.6)	
South	100,000 (39.51)	560 (37.71)	
West	56,000 (21.84)	295 (19.87)	
Urban location (%)	240,000 (92.87)	1,370 (92.26)	0.67
Teaching hospital	190,000 (73.03)	1,120 (75.42)	0.36

Patients with a history of ischemic stroke were significantly older, with a mean of 72.35 years (95% CI: 71.00-73.69) compared to 66.55 years (95% CI: 66.35-66.75) (p < 0.01). The proportion of female patients was similar between the groups (43.1% vs. 45.13%, p = 0.49).

Racial distribution did not significantly differ (p = 0.07), with White patients comprising the majority (67.37% stroke vs. 67.93% no stroke). Median household income distribution also did not differ significantly (p = 0.21). However, insurance status varied significantly (p < 0.01), with Medicare coverage being more common among patients with a history of ischemic stroke (76.12% vs. 63.56%), while Medicaid (9.0% vs. 14.37%) and private insurance (10.38% vs. 17.65%) were less frequent.

Hospital characteristics, including region (p = 0.34), urban location (p = 0.67), and teaching hospital status (p = 0.36), were similar between groups. However, patients with a history of ischemic stroke had a significantly elevated Charlson Comorbidity Index score (p < 0.01), with 86.53% having a score ≥3 compared to 52.87% in the non-stroke group, indicating a greater overall burden of comorbidities.

These findings indicate that patients with a history of ischemic stroke admitted with non-variceal GI bleeding are older, have a more substantial burden of comorbid conditions, and are more likely to be covered by Medicare, which may influence clinical management and outcomes.

Among the 259,025 patients admitted with a principal diagnosis of non-variceal upper GI bleeding, 5,430 (2.1%) died during hospitalization. Mortality occurred in 210 of 1,485 patients (14.14%) who had a prior history of ischemic stroke compared to 5,220 of 257,540 patients (2.03%) without a history of stroke.

Patients with a history of ischemic stroke had a significantly elevated risk of in-hospital mortality, with an unadjusted OR of 7.51 (95% CI: 5.42-10.39; p < 0.01). After adjusting for age, sex, race, median household income, insurance type, hospital region, urban versus rural location, teaching hospital status, and Charlson Comorbidity Index score, the association remained statistically significant, with an adjusted OR of 4.96 (95% CI: 3.47-7.09; p < 0.01).

These results indicate that a history of ischemic stroke is associated with an increased in-hospital mortality among patients admitted with non-variceal GI bleeding, as detailed in Table 3.

**Table 3:** Primary outcome

Outcome	Group	Deaths, n (%)	Odds ratio (OR)	95% confidence interval (CI)	p-value
In-hospital mortality	Ischemic stroke (n = 1,485)	210 (14.14%)	7.51	5.42-10.39	<0.01
	No ischemic stroke (n = 257,540)	5,220 (2.03%)	Reference	–	–
Adjusted mortality	Ischemic stroke	–	4.96	3.47-7.09	<0.01

Secondary outcome

The mean LOS for patients admitted with a principal diagnosis of non-variceal GI bleeding was 4.67 days. After adjusting for confounders, patients with ischemic stroke had an average hospital stay that was 5.68 days longer than those without a history of stroke (95% CI: 4.61-6.76; p < 0.01).

The average total hospital charges for patients admitted with non-variceal GI bleeding were $61,836. After adjustment, hospital charges for patients with ischemic stroke were $100,211.7 higher compared to those without ischemic stroke (95% CI: $77,435.08-$122,988.3; p < 0.01).

Regarding discharge disposition, patients with ischemic stroke had significantly lower odds of discharge home (OR: 0.23; 95% CI: 0.18-0.30; p < 0.01) and increased likelihood of discharge to a skilled nursing facility (OR: 3.30; 95% CI: 2.54-4.30; p < 0.01).

Table 4 presents a detailed comparison of secondary outcomes, including hospital LOS, total hospital charges, and discharge disposition among patients with and without a history of ischemic stroke.

**Table 4:** Secondary outcomes

Adjusted secondary outcomes	Effect estimate for ischemic stroke	95% confidence interval (CI)	p-value
Mean difference in length of stay	5.68 days longer	4.61-6.76 days	<0.01
Mean difference in total hospital charges	$100,212 higher	$77,435-$122,988	<0.01
Odds of discharge home	0.23	0.18-0.30	<0.01
Odds of discharge to skilled nursing facility	3.30	2.54-4.30	<0.01

## Discussion

The NIS database contains 33.3 million observations. According to the 2021 dataset, 259,025 patients were admitted to the hospital with a primary diagnosis of non-variceal upper GI bleeding. Of these, only 0.57% had a prior history of ischemic stroke. Patients with a prior history of stroke who presented with GI bleeding had a higher mean age of 72.35 years compared to patients without stroke, and this difference was statistically significant. These patients also had a significantly greater burden of comorbid conditions at the time of presentation.

Race, median household income, geographical location, and local hospital availability were not statistically significant in the analysis.

Acute upper GI bleeding is a serious complication in patients with a prior history of stroke, with most studies estimating its occurrence between 1% and 5% [17,18]. However, the incidence in our dataset was below 1%.

Patients in this cohort, those with GI bleeding and prior stroke, were generally older, had a higher comorbidity burden, and often presented with altered levels of consciousness [19-21]. Many of these patients also had a prior history of GI bleeding due to the use of medications such as NSAIDs and anticoagulants, which predispose them to recurrent bleeding episodes [22]. Their higher comorbidity burden increases the likelihood of polypharmacy, impaired consciousness, *Helicobacter ​​​​​​pylori* infection, and neurological factors that contribute to stress ulcer formation [23-25]. Additionally, many patients had a history of atrial fibrillation, which is independently associated with GI bleeding [26].

Patients with non-variceal upper GI bleeding and a prior history of stroke demonstrated a higher risk of in-hospital mortality, likely due to advanced age, multiple comorbidities, and altered mental status [27,28].

Our statistical analysis was adjusted for age, sex, median household income, insurance type, hospital location (urban vs. rural), and comorbidity. The results remained statistically significant, with an OR of 4.96 (95% CI: 3.47-7.09; p < 0.01). These findings suggest that a prior history of stroke is an independent predictor of poor outcomes in patients presenting with non-variceal GI bleeding.

Secondary outcomes also demonstrated longer hospital stays, higher hospital charges, and increased medical care requirements at the time of discharge for patients with a prior history of stroke. Although this group represents a small subset of patients with upper GI bleeding, they tend to be older, more medically complex, and require more intensive care.

Patients with a prior history of stroke, depending on the severity of their neurological deficits, comorbidities, and mental status, require a higher level of attention and individualized management.

Limitations

This study has several limitations. First, as it relies on data from the NIS database, which consists of administrative discharge-level records, it assumes accurate coding of diagnoses and procedures. However, the potential for misclassification cannot be entirely ruled out. Although the NIS represents approximately 20% of all hospital discharges and employs stratified sampling with weighting to generate national estimates, it is still a sample and not a complete census. Therefore, certain nuanced patient- or hospital-level characteristics may not be fully captured.

Additionally, the NIS does not include clinical details such as timing of events, medication use, laboratory results, or temporal associations. Lastly, the retrospective nature of this study limits our ability to establish causal relationships between a history of ischemic stroke and the observed outcomes. Future research utilizing prospective study designs and incorporating more detailed clinical variables would be valuable in further elucidating these associations.

## Conclusions

Among patients admitted with a primary diagnosis of acute non-variceal upper GI bleeding, those with a prior history of ischemic stroke were generally older and presented with a greater burden of comorbidities. This subgroup experienced significantly worse clinical outcomes, including higher in-hospital mortality and increased healthcare utilization. The presence of a prior ischemic stroke was found to be an independent predictor of poor prognosis in this population.

These patients also required more intensive inpatient care and were more frequently discharged to facilities providing extended post-acute care. The findings highlight the need for heightened clinical vigilance and multidisciplinary management strategies in patients with a history of stroke who present with upper GI bleeding.
